# Supplementary material for: Emergent ribozyme behaviors in oxychlorine brines indicate a unique niche for molecular evolution on Mars
Source: Nat Commun. 2024 May 20;15:3863. doi: 10.1038/s41467-024-48037-2 (PMC11106070; doi:10.1038/s41467-024-48037-2)
Supplement: Supplementary file 1 — Supplementary Information [file 41467_2024_48037_MOESM1_ESM.pdf]

Supplementary Information

for

Emergent ribozyme behaviors in oxychlorine brines indicate a unique niche for molecular evolution on Mars

Tanner G. Hoog, Matthew R. Pawlak, Nathaniel J. Gaut, Gloria C. Baxter, Thomas A. Bethel, Katarzyna P. Adamala, Aaron E. Engelhart

Contents:

Supplementary Figure 1. All uncropped gel images.

Supplementary Figure 2. Hammerhead ribozyme and EcoRI enzyme rate constants as a function of perchlorate.

Supplementary Figure 3. RNase HII kinetics in perchlorate solutions.

Supplementary Figure 4. Broccoli aptamer melting curves in perchlorate solutions.

Supplementary Figure 5. TaqI-v2 and HaBlap kinetics and rate constants in perchlorate solutions.

Supplementary Figure 6. Hammerhead ribozyme activity in solutions of urea with hexamer interference.

Supplementary Figure 7. rPS2.M-heme stoichiometry and Amplex Red oxidation.

Supplementary Figure 8. MCD Chlorination by PS2.M/heme and HRP with hypochlorite and chlorite in perchlorate solutions.

Supplementary Figure 9. Characterization of heme turnover by rPS2.M/heme.

Supplementary Figure 10. Heme requires rPS2.M to efficiently catalyze chlorination.

Supplementary Figure 11. Extinction coefficient of hydrolyzed nitrocefin as a function of salt concentration.

Supplementary Table 1. List of oligonucleotides used in this study.

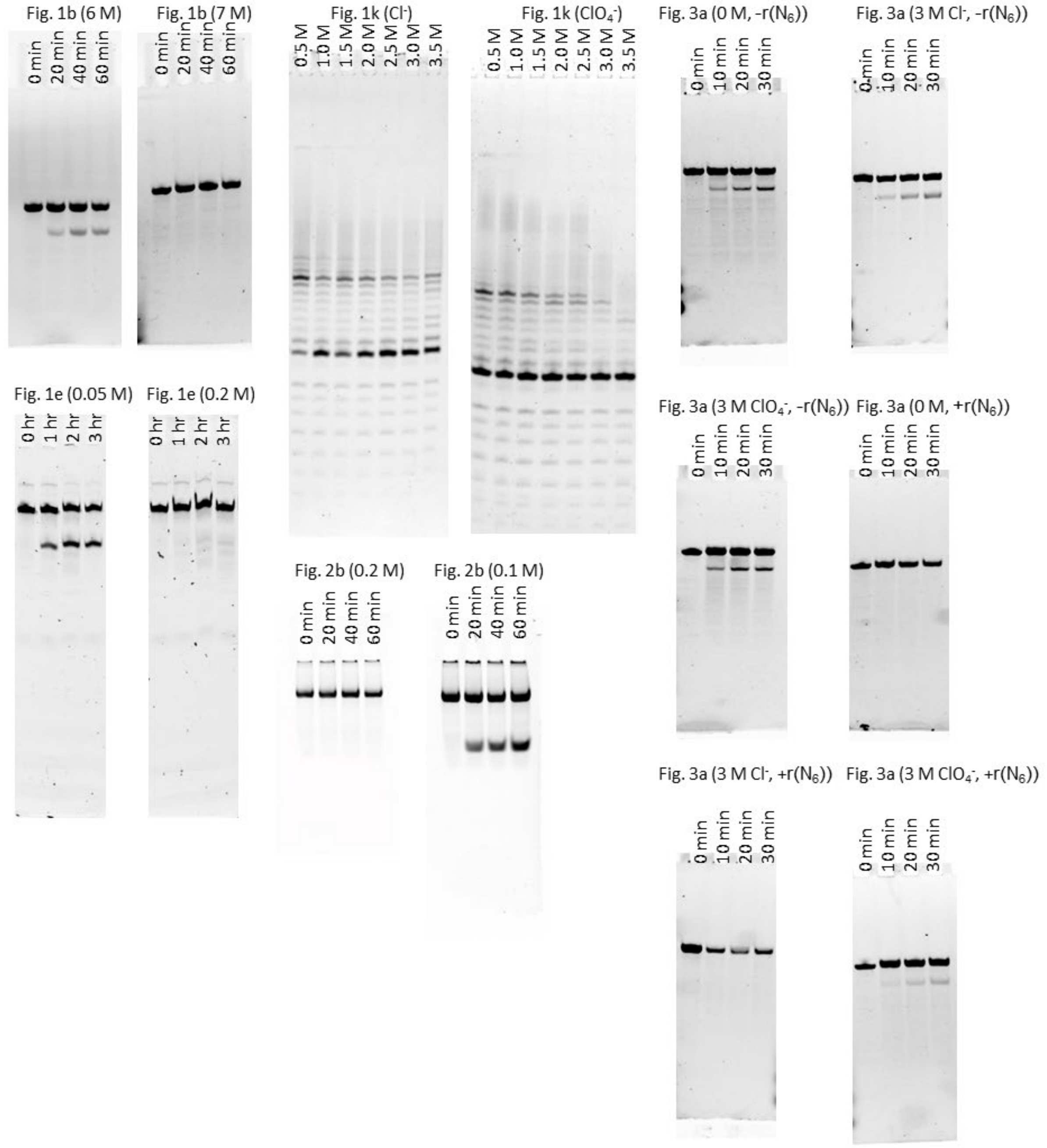

Supplementary Figure 1. All uncropped gel images.

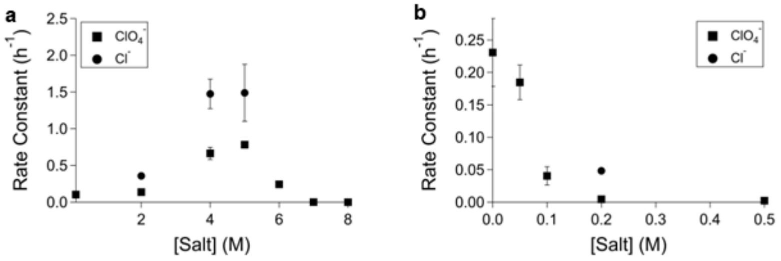

Supplementary Figure 2. Hammerhead ribozyme and EcoRI enzyme rate constants as a function of perchlorate. Rate constant as a function of salt concentration for (**a**) the hammerhead ribozyme (**b**) and EcoRI. Error bars represent the standard error of the mean with n=3.

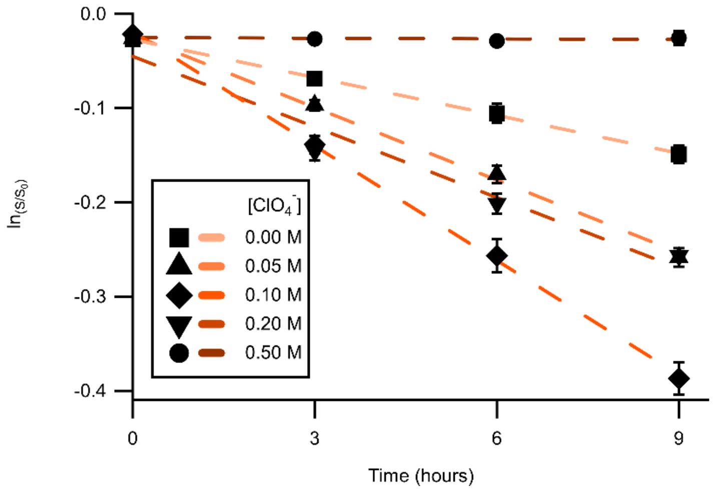

Supplementary Figure 3. RNase HII kinetics in perchlorate solutions. Error bars represent the standard error of the mean with n=3.

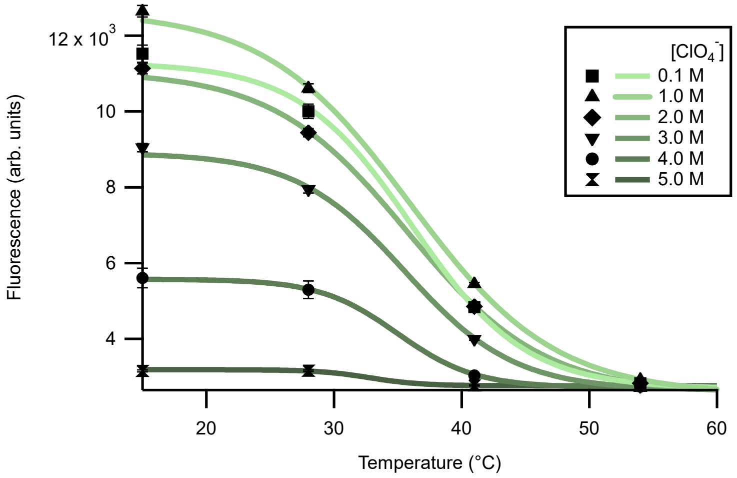

Supplementary Figure 4. Broccoli aptamer melting curves in perchlorate solutions. Markers are used sparsely every 10 data points for clarity. The lines are sigmoidal curves fitted to the data using Igor Pro 9. Error bars represent the standard error of the mean with n=3.

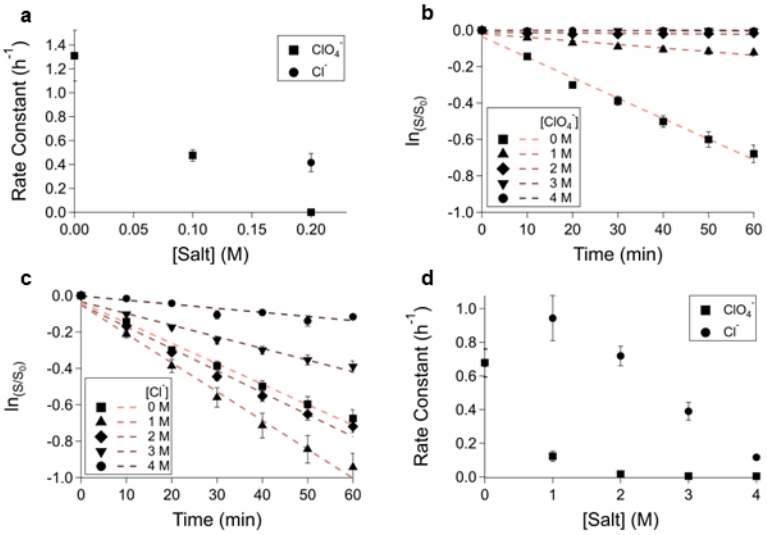

Supplementary Figure 5. TaqI-v2 and HaBlap kinetics and rate constants in perchlorate solutions. a, TaqI-v2 rate constant as a function of salt concentration. HaBlap catalyzed nitrocefin hydrolysis in (**b**) perchlorate and (**c**) chloride solutions. **d,** HaBlap rate constant as a function of salt concentration. Error bars represent the standard error of the mean with n=3.

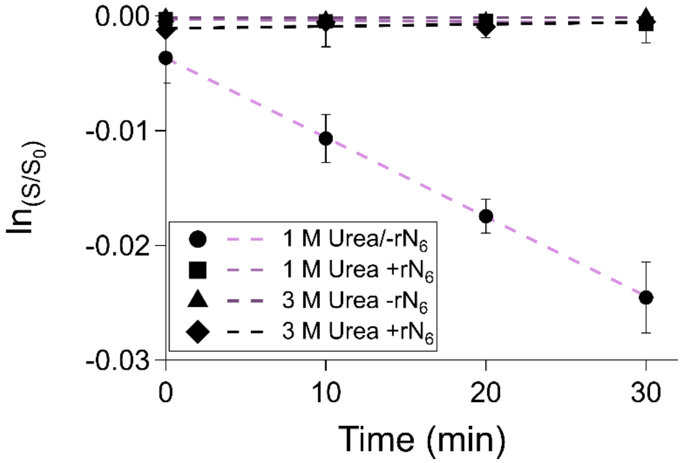

Supplementary Figure 6. Hammerhead ribozyme activity in solutions of urea with hexamer interference. Error bars represent the standard error of the mean with n=3.

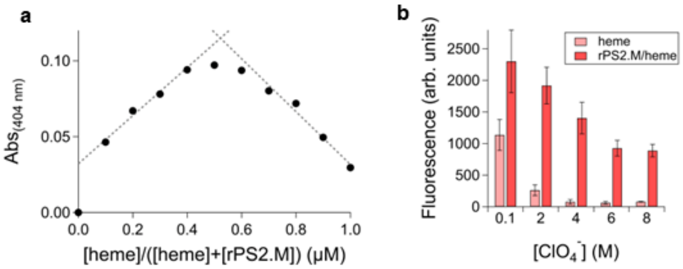

Supplementary Figure 7. rPS2.M-heme stoichiometry and Amplex Red oxidation. a, The rPS2.M G quadruplex interacts 1:1 with heme as shown using Job's method of continuous variation. **b,** The rPS2.M/heme holoenzyme performs peroxidation cycles with hydrogen peroxide and oxidizing Amplex Red to resorufin. Error bars represent the standard error of the mean with n=3.

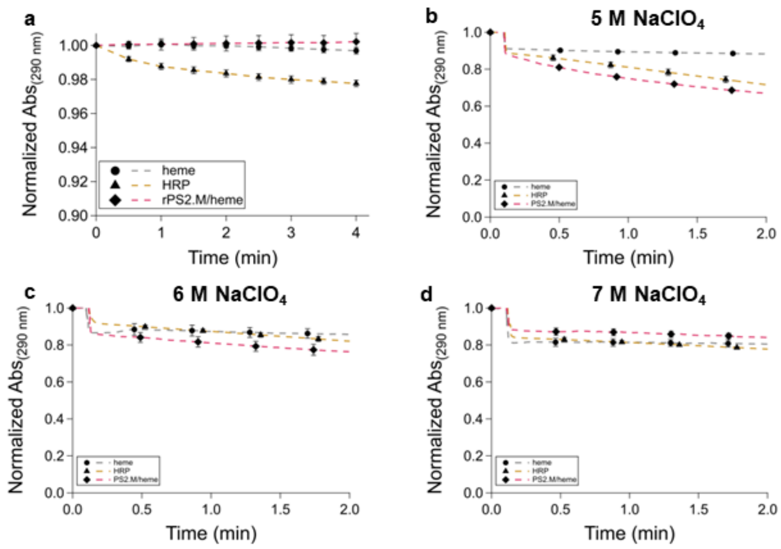

Supplementary Figure 8. MCD Chlorination by PS2.M/heme and HRP with hypochlorite and chlorite in perchlorate solutions. a, MCD chlorination with heme, rPS2.M/heme, and HRP using sodium hypochlorite instead of sodium chlorite proceeds with HRP, but not heme or rPS2.M/heme. **b-d,** MCD chlorination with heme, rPS2.M/heme, and HRP in 5 M (**b**), 6 M (**c**), and 7 M (**d**) sodium perchlorate solutions. Error bars represent the standard error of the mean with n=3.

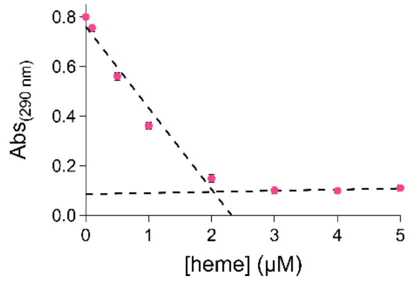

Supplementary Figure 9. Characterization of heme turnover by rPS2.M/heme. MCD (50 μM) chlorination with sodium chlorite (200 μM) catalyzed by rPS2.M (7.5 μM) and varying amounts of heme. Based on chlorinating 50 μM MCD with 2 μM heme in limiting heme conditions, and the requirement for two oxidations of heme by chlorite per chlorination event, this gives a turnover number of 12.5 reactions/heme. Error bars represent the standard error of the mean with n=3.

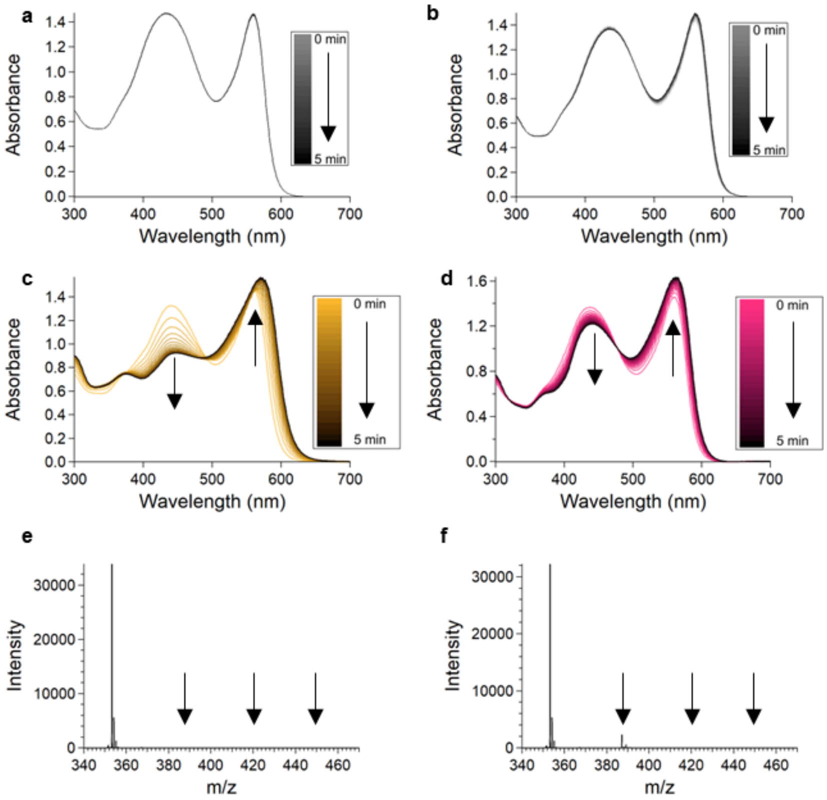

Supplementary Figure 10. Heme requires rPS2.M to efficiently catalyze chlorination. Phenol red chlorination by sodium chlorite in buffer containing no heme (**a**), buffer containing heme (**b**), buffer containing HRP (**c**), and buffer containing rPS2.M/heme (**d**) monitored by visible light absorbance. Phenol red chlorination by sodium chlorite catalyzed by buffer containing no heme (**e**) and buffer containing heme (**f**) monitored by ESI-MS. Heme only catalyzes marginal chlorination of phenol red to chlorophenol red without the addition of rPS2.M (as shown in **Fig. 4h**). Expected molecular weights: 353.05, 387.01, 420.97, and 454.93. Heme only shows a 353.1 peak and trace 387.0 peak.

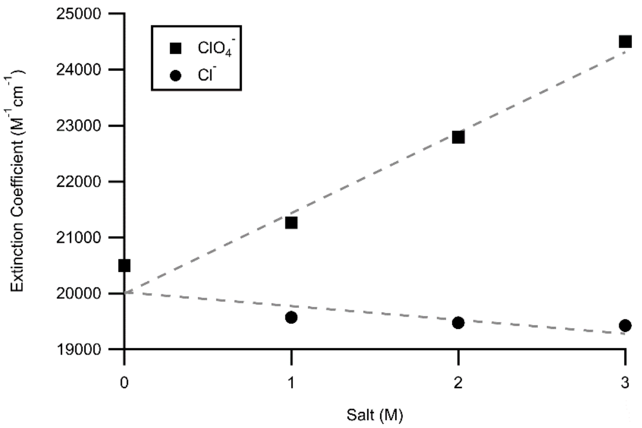

Supplementary Figure 11. Extinction coefficient of hydrolyzed nitrocefin as a function of salt concentration. Error bars represent the standard error of the mean with n=3.

Oligonucleotide	RNA/DNA	Sequence
EcoRI cleavage assay strand A	DNA	/56-FAM/CGT GTC AGT GAA TTC TTC GAG ATC
RNase HII cleavage assay Strand A	Mixed DNA/RNA	/56-FAM/CGT GTC AGT rGAA TTC TTC GAG ATC
EcoRI/RNAse HII cleavage assay Strand B	DNA	GAT CTC GAA GAA TTC ACT GAC ACG
TaqI-v2 cleavage assay (sense)	DNA	/56-FAM/TCA TAC CTA TTC GAT TGG ATC CTT CCG GTT GGC CGT TAC GGC CTT GCG CTT GAA TTC CGT GCA CAG AA
TaqI-v2 cleavage assay (antisense)	DNA	TTC TGT GCA CGG AAT TCA AGC GCA AGG CCG TAA CGG CCA ACC GGA AGG ATC CAA TCG AAT AGG TAT GA
Hammerhead ribozyme strand A	RNA	/56-FAM/CGC GCC GAA ACA CCG UGU CUC GAG C
Hammerhead ribozyme strand B	RNA	GGC UCG ACU GAU GAG GCG CG
Hammerhead displacement strand	DNA	CGC GCC TCA TCA GTC GAG CC
Broccoli T7 template (sense)	DNA	**TAA TAC GAC TCA CTA TAG** GAG ACG GTC GGG TCC AGA TAT TCG TAT CTG TCG AGT AGA GTG TGG GCT C
Broccoli T7 template (antisense)	DNA	GAG CCC ACA CTC TAC TCG ACA GAT ACG AAT ATC TGG ACC CGA CCG TCT CCT ATA GTG AGT CGT ATT A
Broccoli aptamer	RNA	GGA GAC GGU CGG GUC CAG AUA UUC GUA UCU GUC GAG UAG AGU GUG GGC UC
tC19Z T7 template (sense)	DNA	**TAA TAC GAC TCA CTA TAG** TCA TTG AAA AAA AAA GAC AAA TCT GCC CTC AGA GCT TGA GAA CAT CTT CGG ATG CAG AGG AGG CAG CCT TCG GTG GCG CGA TAG CGC CAA CGT TCT CAA CAG ACA CCC AAT ACT CCC GCT TCG GCG GGT GGG GAT AAC ACC TGA CGA AAA GGC GAT GTT AGA CAC GCC CAG GTC ATA ATC CCC GGA GCT TCG GCT CC
tC19Z T7 template (antisense)	DNA	GUC AUU GAA AAA AAA AGA CAA AUC UGC CCU CAG AGC UUG AG AAC AUC UUC GGA UGC AGA GGA GGC AGC CUU CGG UGG CGC GAU AGC GCC AAC GUU CUC AAC AGA CAC CCA AUA CUC CCG CUU CGG CGG GUG GGG AUA ACA CCU GAC GAA AAG GCG AUG UUA GAC ACG CCC AGG UCA UAA UCC CCG GAG CUU CGG CUC C
tC19Z ribozyme		GUC AUU GAA AAA AAA AGA CAA AUC UGC CCU CAG AGC UUG AGA ACA UCU UCG GAU GCA GAG GAG GCA GCC UUC GGU GGC GCG AUA GCG CCA ACG UUC UCA ACA GAC ACC CAA UAC UCC CGC UUC GGC GGG UGG GGA UAA CAC CUG ACG AAA AGG CGA U
tC19Z Template RNA	RNA	GUC AAU GAC ACG CUU CGC ACG GUU GGC AGA AAA AAA AAA
tC19Z RNA primer	RNA	/56-FAM/CTG CCA ACC G
rPS2.M G quadruplex	RNA	GUG GGU AGG GCG GGU UGG

T7 promoter sequences are bolded. RNA primer binding sequences for RNA polymerase assays are underlined.

Supplementary Table 1. List of oligonucleotides used in this study.
